# Adolescent Body Mass Index, Weight Trajectories to Adulthood, and Osteoporosis Risk

**DOI:** 10.1001/jamanetworkopen.2025.25079

**Published:** 2025-08-04

**Authors:** Maya Simchoni, Regev Landau, Estela Derazne, Orit Pinhas-Hamiel, Afif Nakhleh, Inbal Goldshtein, Avishai M. Tsur, Arnon Afek, Gabriel Chodick, Liana Tripto-Shkolnik, Gilad Twig

**Affiliations:** 1 Tzameret Department of Military Medicine and Faculty of Medicine, The Hebrew University of Jerusalem, Jerusalem, Israel; 2 Israel Defense Forces Medical Corps, Ramat Gan, Israel; 3 Endocrine Institute, Shamir Medical Center, Beer Yaakov, Israel; 4 School of Medicine, Gray Faculty of Medical and Health Sciences, Tel Aviv University, Tel Aviv, Israel; 5 Pediatric Endocrinology and Diabetes Unit, Edmond and Lily Safra Children’s Hospital, Sheba Medical Center, Ramat Gan, Israel; 6 Institute of Endocrinology, Diabetes and Metabolism, Rambam Health Care Campus, Haifa, Israel; 7 Diabetes and Endocrinology Clinic, Maccabi Healthcare Services, Haifa, Israel; 8 Maccabitech Institute for Research and Innovation, Maccabi Healthcare Services, Tel Aviv, Israel; 9 Department of Medicine, Sheba Medical Center, Ramat Gan, Israel; 10 Dina Recnati School of Medicine, Reichman University, Herzliya, Israel; 11 The Gertner Institute for Epidemiology and Health Policy Research, Sheba Medical Center, Ramat Gan, Israel; 12 Division of Endocrinology, Diabetes and Metabolism, Sheba Medical Center, Ramat Gan, Israel; 13 Department of Epidemiology and Preventive Medicine, School of Public Health, Gray Faculty of Medical and Health Sciences, Tel Aviv University, Tel Aviv, Israel

## Abstract

**Importance:**

There are limited data regarding adolescent weight among healthy individuals and their trajectory through early adulthood with respect to bone health.

**Objective:**

To assess the association between adolescent body mass index (BMI) and osteoporosis risk while accounting for BMI change during early adulthood.

**Design, Setting, and Participants:**

A retrospective population-based cohort study from 1967 to 2019. Participants were Israeli-born adolescents aged 16 to 19 years who were evaluated for military service. Data were analyzed from January 2023 to March 2025.

**Exposure:**

Weight and height were measured to calculate BMI at adolescence, and additional sociodemographic and medical data were collected. Health status at baseline and incident cancer and diabetes throughout adulthood were strictly controlled.

**Main Outcomes and Measures:**

Osteoporosis diagnosis until 2022, recorded in the osteoporosis registry of Maccabi Healthcare Services (the second-largest Israeli health care system). Cox proportional hazard models were applied. Adult BMI measurement was available for 74% of the study population and was used to assess the association between adolescence-to-adulthood weight trajectory and incident osteoporosis.

**Results:**

In this cohort study of 1 083 491 adolescents, 21 497 (4.58%) women and 6929 (1.13%) men were enrolled in the osteoporosis registry during a cumulative follow-up of 19 400 208 person-years (mean [SD] age at follow-up, 23.7 [8.5] years). There was a consistent inverse association between adolescent BMI and osteoporosis risk in adulthood. The crude incidence rate of osteoporosis decreased from 330.2 per 100 000 person-years among those with extreme underweight (<3rd percentile) to 78.9 among those with obesity (≥95th percentile). Adjusted hazard ratios for osteoporosis ranged from 1.88 (95% CI, 1.74–2.04) to 0.83 (95% CI, 0.77–0.89) in women and from 1.82 (95% CI, 1.64–2.01) to 1.04 (95% CI, 0.93–1.16) in men, using normal BMI as the reference. A sex-specific difference in osteoporosis risk was notable, with obesity not showing a protective association in men compared with women. The findings were robust across multiple models and sample restrictions, and the highest risk was observed in individuals who remained underweight from adolescence into adulthood.

**Conclusions and Relevance:**

In this cohort study, BMI at a young age and its trajectory to adulthood were significantly associated with risk for osteoporosis in adult life.

## Key Points

**Question:**

What is the association between adolescent body mass index (BMI) and osteoporosis risk while accounting for BMI change during early adulthood?

**Findings:**

This cohort study of 1.1 million Israeli adolescents found a direct inverse association between adolescent BMI for osteoporosis incidence, which persisted in various models adjusted for sociodemographic confounders and preexisting illnesses. The highest risk was observed in those remaining underweight from adolescence to adulthood, while weight gain from underweight was associated with reduced risk.

**Meaning:**

These findings suggest that BMI at a young age and its trajectory to adulthood are associated with risk for osteoporosis in adult life.

## Teaser

This cohort study evaluates the association between adolescent body mass index (BMI) and osteoporosis risk, while accounting for BMI change during early adulthood.

## Introduction

Osteoporosis is estimated to affect 200 million people worldwide^1^ and poses an important public health issue due to its lifetime prevalence,^1^ high economic burden,^2^ and the adverse outcomes of osteoporotic fractures.^3,4^ Evidence for the negative effect of low body mass index (BMI) on different bone health parameters, including bone mineral density (BMD),^5,6^ fracture risk^5,7,8^ and risk for osteoporosis,^5,7,9^ are abundant. However, evidence regarding the effect of BMI at a young age on bone health in later life is insufficient, and existing studies lack data regarding comorbid conditions with a potential effect on bone health. The goal of this research is to examine the association between BMI at adolescence, adolescent-adult BMI trajectory, and the risk of osteoporosis in adulthood.

## Methods

### Study Population

This retrospective cohort study included all Israeli adolescents aged 16 to 19 years who were examined for mandatory military service from January 1, 1967, to December 31, 2019 (1 134 940 adolescents), and were members of Maccabi Healthcare Services (MHS) (eFigure in Supplement 1). Excluded from the cohort were people with missing baseline BMI data (26 407 participants [2.33%]) and those with preexisting comorbidities that are linked with low bone density and/or higher risk for osteoporosis (25 042 participants [2.21%]), including people with autoimmune diseases (3982 participants [0.35%]), parathyroid and calcium homeostasis disorders (140 participants [0.01%]), and any cancer history (1411 participants [0.12%]) (eFigure in Supplement 1). The final study sample included 1 083 491 persons (614 584 men [56.7%]) with mean (SD) age of 17.3 (0.5) years. The Israel Defense Forces Medical Corps institutional review board ethically approved this study and waived the requirement for written informed consent. This report follows the Strengthening the Reporting of Observational Studies in Epidemiology (STROBE) reporting guideline for cohort studies.

### Adolescent Data Collection and Study Variables

One year before mandatory military service, Israeli adolescents attend a prerecruitment evaluation process including medical, cognitive, and sociodemographic assessments. The medical evaluation includes a review of medical history by a military physician based on detailed reports delivered by the primary care physician. The medical examination includes weight and height measurements (in light clothing and barefoot). BMI was calculated by dividing the weight in kilograms (rounded to the nearest 0.5 kg) by the height squared in meters (rounded to 1 cm). BMI was categorized into 7 groups based on age- and sex-specific percentiles of the US Centers for Disease Control and Prevention, which were previously validated for Israeli adolescents^10^: below the 3rd percentile (extreme underweight), 3rd to 4th percentile (underweight), 5th to 9th percentile (low weight), 10th to 49th percentile (low-normal BMI), 50th to 84th percentile (high-normal BMI), 85th to 94th (overweight), and above the 95th (obesity). Further referrals to medical tests and/or consultations with specialists were performed as needed. Adolescents were classified as having unimpaired health if their medical assessment revealed absence of medical conditions requiring a chronic medical treatment and/or follow-up, and no history of cancer or major operations. Cognitive assessment included a general intelligence test that is comparable with the intelligence quotient (IQ)^11^ and has high external validity.^12^ This score was transformed into annual and sex-specific *z* scores, based on all examinees in a given calendar year, and categorized into low (lower than −1 *z* score), medium (between −1 *z* score to 1 *z* score), and high (higher than 1 *z* score). This score has been shown to be closely related to various cardiometabolic conditions, such as type 2 diabetes.^13,14^ Sociodemographic data were routinely transferred to military authorities by various governmental ministries. Years of schooling were reported by the Ministry of Education and were dichotomized (≥11 vs <11 years).^15^ The Israeli Central Bureau of Statistics scoring system was used to determine residential socioeconomic status (SES) on a scale of 1 to 10, based on residential municipality.^16^ This variable was categorized into low (1-4), medium (5-7) and high (8-10), as used previously.^17^ Country of birth was reported by the Israeli Ministry of Interior and was dichotomized by whether the examinee was born in Israel.

### Osteoporosis Registry at MHS

Upon completion of military service (and before that, from birth until recruitment), as part of the National Health Insurance Law, every Israeli citizen receives medical care from 1 of the 4 health care systems. The second largest of them is MHS, which insures approximately 28% of the Israeli population.^18^ The primary outcome of the study was incident osteoporosis diagnosed after military service as recorded in MHS osteoporosis registry.^19^ The follow-up period was defined from the later of either of January 1, 1998 (the establishment of the osteoporosis registry), or the prerecruitment assessment, or until onset of osteoporosis, death, or data lock on August 31, 2022. Data from the osteoporosis registry were linked to the Israel Defense Forces database using the participants’ national identification number to enable the linkage of medical and socioeconomic data collected at adolescence. Osteoporosis was reported to the registry when a participant met 1 of the following criteria: (1) femoral neck or lumbar BMD *T* score of −2.5 or less; (2) osteoporosis-related major bone fracture: Colles, humerus, hip, or vertebral fracture; (3) or 2 or more purchases of antiosteoporotic medications. Date of osteoporosis diagnosis was determined as the earliest of the previously mentioned conditions. It is noteworthy that while all individuals recorded in the osteoporosis registry were positive for at least 1 of the previously mentioned criteria, the specific criterion for entering the registry was unavailable to us. Measurements of weight and height at adulthood—at the diagnosis time point—were obtained as part of routine clinical visits. From 2010, documentation of measured weight and height in the electronic medical record was considered a quality measure in medical service.^20^

### Statistical Analysis

Analyses were dichotomized by sex determined at birth. Differences in baseline characteristics of the study groups were assessed using analysis of variance with *F* tests for continuous variables and χ^2^ tests for categorical variables. Incident rate of osteoporosis was calculated per 10^5^ person-years. Kaplan-Meier curves and log-rank tests were used to assess time to event. Cox proportional hazard models were applied to calculate the hazard ratios (HR) and 95% CIs for incident osteoporosis using the high-normal BMI group as the reference. The basic model was only adjusted for age at study entry, whereas the multivariable model was prespecified to birth year, education, cognitive performance, socioeconomic status, and country of birth. Missing data for covariates in the model amounted to 35 285 cases (3.26%).

We conducted several sensitivity and subgroup analyses: (1) analysis was limited to those with unimpaired health at baseline to minimize confounding by a coexisting medical condition; (2) analysis was limited to people without incident diagnosis of cancer or diabetes during the study follow-up; (3) we stratified the outcome by age of osteoporosis diagnosis, before or after age 52 years, to better account for menopause status; (4) as during the study period Israel has moved from a developing to a developed country with a marked increase in adolescent severe obesity observed in late 1990s,^21^ we limited analysis to examinees who entered the study after 1995 (men) or 1996 (women). This allowed us to better control ambient exposures related to Israel being a developed rather than a developing country (ie., last 2 rather than 5 decades) and to study the association without time gaps between study entrance and availability of the osteoporosis registry; (5) analysis that was limited to those who were permanent MHS members (ie, those with continuous follow-up from study entrance to onset of osteoporosis [491 058 participants], death [394 642 participants], or August 1, 2022) to better isolate possible bias introduced by transition between health care practitioners.

As part of an exploratory analysis, we accounted for the association between adolescence-to-adulthood BMI trajectory and incident osteoporosis. This included the first weight and height measurements that were obtained after military discharge as part of routine clinical visits, as reported previously for gestational diabetes risk.^22^ Differences in baseline characteristics among those with and without adulthood BMI data were examined. Follow-up definition and diagnostic criteria remained the same as the main analysis. We classified BMI status at either adolescence or adulthood into underweight, nonobese BMI, and obesity, thereby defining 9 categories for adolescent-to-adulthood trajectories. We computed the HR for incident osteoporosis in each category, with individuals maintaining a nonobese BMI as the reference group. To assure that association (especially among those with weight loss) was not confounded by cancer, we conducted a sensitivity analysis that excluded those with incident cancer during follow-up. All statistical tests were 2-tailed, and *P* < .05 was considered statistically significant. Statistical analyses were performed using SPSS, version 25.0 (IBM) from January 2023 to March 2025.

## Results

Among 1 083 491 adolescents, 21 497 (4.58%) women and 6929 (1.13%) men were enrolled in the osteoporosis registry. A total of 65 244 (6.02%) were underweight, of which 43 001 (3.97%) were extremely underweight below the 3rd percentile. A total of 53 586 (4.94%) were between the 5th to 9th percentile, 821 348 (75.77%) had normal weight (divided into low-normal and high-normal), 97 801 (9.02%) were overweight, and 45 512 (4.20%) had obesity. There was an interaction between sex and adolescent BMI and the study outcome (HR among male adolescents with obesity, 1.17; 95% CI, 0.99-1.38 vs women, 0.83; 95% CI, 0.71-0.96; *P *for interaction < .001). Baseline characteristics of the study population according to adolescent BMI groups are presented in Table 1. For both sexes, the proportions of low residential socioeconomic status and full education generally increased with heavier BMI categories.

**Table 1.** Baseline Characteristics of the Study Population According to Adolescent Body Mass Index (BMI)^a^

Characteristic	Participants in each percentile, No. (%)						
	<3rd	3rd to 4th	5th to 9th	10th to 49th	50th to 84th	85th to 94th	≥95th
Men	30 201 (4.91)	14 608 (2.38)	33 842 (5.51)	247 801 (40.30)	202 605 (32.95)	54 081 (8.80)	31 446 (5.12)
BMI, mean (SD)^b^	16.8 (0.7)	17.7 (0.3)	18.2 (0.3)	20.0 (0.9)	22.9 (1.1)	26.5 (1.0)	31.7 (3.1)
Weight, mean (SD), kg	50.8 (4.6)	53.7 (4.3)	55.3 (4.4)	60.9 (5.4)	69.8 (6.3)	80.7 (7.0)	96.6 (12.2)
Height, mean (SD), cm	173.9 (7.1)	173.9 (6.8)	174.0 (6.7)	174.1 (6.7)	174.3 (6.7)	174.5 (6.9)	174.5 (7.2)
Age at BMI measurement, mean (SD), y	17.4 (0.5)	17.4 (0.5)	17.4 (0.5)	17.3 (0.5)	17.3 (0.5)	17.3 (0.5)	17.3 (0.5)
Age at follow-up beginning, mean (SD), y	24.1 (8.2)	24.6 (8.5)	24.7 (8.6)	24.8 (9.0)	24.0 (8.9)	22.3 (8.1)	20.4 (6.5)
Full education	25 671 (85)	12 562 (86)	28 766 (86)	215 587 (86)	180 319 (89)	48 132 (89)	27 987 (89)
Socioeconomic status^c^							
Low	6946 (23)	3360 (23)	7445 (22)	52 038 (21)	40 521 (20)	11 898 (22)	7862 (25)
Medium	14 798 (49)	7158 (49)	16 583 (49)	123 901 (50)	103 309 (51)	28 122 (52)	16 665 (53)
High	8457 (28)	4090 (28)	9814 (29)	71 862 (29)	58 775 (29)	14 061 (26)	6919 (22)
Cognitive score^d^							
Low	5436 (18)	2337 (16)	5076 (15)	32 214 (13)	24 313 (12)	8112 (15)	6604 (21)
Medium	20 236 (67)	9787 (67)	23 014 (68)	168 706 (68)	139 797 (69)	37 311 (69)	20 754 (66)
High	4529 (15)	2484 (17)	5752 (17)	47 081 (19)	38 5495 (19)	8653 (16)	4088 (13)
Israeli born	24 766 (82)	11 979 (82)	27 750 (82)	203 097 (82)	166 136 (82)	44 276 (82)	26 100 (83)
Women	12 800 (2.73)	7635 (1.63)	19 744 (4.21)	190 915 (40.73)	180 027 (38.40)	43 720 (9.32)	14 066 (2.98)
BMI, mean (SD)^b^	16.3 (0.6)	17.1 (0.2)	17.7 (0.2)	19.6 (0.9)	22.7 (1.2)	27.0 (1.2)	33.0 (3.0)
Weight, mean (SD), kg	43.8 (3.6)	45.8 (3.5)	47.0 (3.6)	51.9 (4.4)	59.8 (5.5)	71.0 (6.4)	87.1 (10.6)
Height, mean (SD), cm	163.8 (6.5)	163.3 (6.2)	163.0 (6.1)	162.6 (6.0)	162.1 (6.1)	162.1 (6.3)	162.5 (6.4)
Age at BMI measurement, mean (SD), y	17.4 (0.5)	17.3 (0.5)	17.3 (0.4)	17.3 (0.4)	17.3 (0.4)	17.2 (0.4)	17.2 (0.4)
Age at follow-up beginning, mean (SD), y	22.7 (7.7)	23.1 (7.9)	23.3 (8.0)	23.6 (8.3)	23.2 (8.2)	21.7 (7.3)	19.7 (5.4)
Full education	12 160 (95)	7330 (96)	18 954 (96)	183 478 (96)	172 826 (96)	41 971 (96)	13 503 (96)
Socioeconomic status^c^							
Low	2048 (16)	1222 (16)	2962 (15)	28 637 (15)	28 804 (16)	7432 (17)	2532 (18)
Medium	6656 (52)	3969 (52)	10 464 (53)	99 276 (52)	93 614 (52)	24 046 (55)	8013 (57)
High	4096 (32)	2444 (32)	6318 (32)	62 002 (33)	57 609 (32)	12 242 (28)	3521 (25)
Cognitive score^d^							
Low	1664 (13)	839 (11)	1974 (10)	15 273 (8)	16 202 (9)	5684 (13)	2532 (18)
Medium	9472 (74)	5804 (76)	15 005 (76)	143 186 (75)	135 020 (75)	32 790 (75)	10 406 (74)
High	1664 (13)	992 (13)	2765 (14)	32 456 (17)	28 805 (16)	5246 (12)	1128 (8)
Israeli born	10 496 (82)	6337 (83)	16 583 (84)	160 369 (84)	151 223 (84)	36 725 (84)	11 815 (84)

^a^ Note that since follow-up could start from the time the MHS osteoporosis registry was established (January 1998), examinees with higher BMI categories who were more likely to enter the study in later decades had a slightly younger age at beginning of follow-up.

^b^ Body mass index calculated as weight in kilograms divided by height in meters squared.

^c^ Low indicates x, medium indicates x, and high indicates x.

^d^ Low indicates x, medium indicates x, and high indicates x.

The current study population, which was based on people insured by MHS, had similar health-related indexes at baseline vs those who underwent the screening evaluation but were insured by other health care systems, with comparable BMI and blood pressure values and levels of unimpaired health (eTable 1 in Supplement 1). Notably, high residential socioeconomic status and cognitive performance were more prevalent among those insured by MHS. Baseline characteristics of the participants who were lost to follow-up due to a transfer to another health care practitioner were also comparable with those who completed follow-up (eTable 2 in Supplement 1).

### Adolescent BMI and Osteoporosis

**Figure 1.**
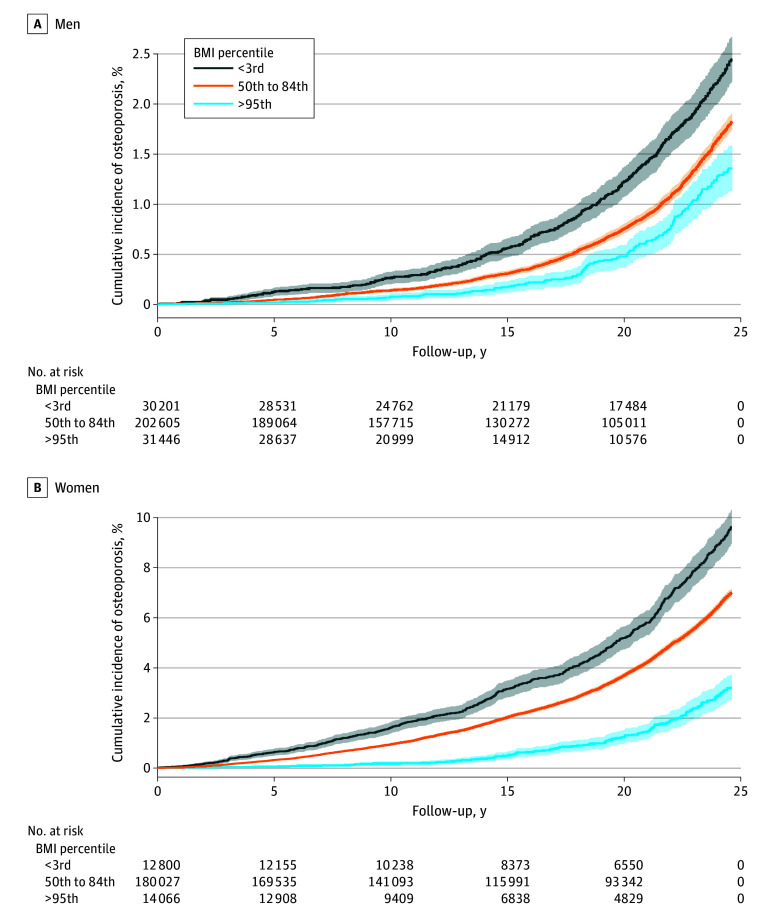
Kaplan-Meier Curves for Incident Osteoporosis Kaplan-Meier curves were plotted for 3 representatives of 7 adolescent BMI groups. BMI indicates body mass index, calculated as weight in kilograms divided by height in meters squared. Shading indicates 95% CIs.

The mean (SD) age at follow-up initiation was 23.7 (8.5) years (Table 1). During 19 400 208 person-years, there were 28 426 incident cases of osteoporosis (Table 2): 21 497 (4.58%) among women and 6929 (1.13%) among men. The mean (SD) age at diagnosis was 54.6 (7.9) years for women and 57.8 (11.3) years for men. The crude incidence rates (events per 10^5^ person-years) across BMI groups for osteoporosis among women were 330.2 (<3rd percentile), 313.9 (3rd to 4th percentile), 325.8 (5th to 9th percentile), 297.5 (10th to 49th percentile), 234.7 (50th to 84th percentile), 150.0 (85th to 94th percentile) and 78.9 (≥95th percentile). A similar pattern was observed in men, albeit with lower absolute rates (Table 2). Kaplan-Meier curves confirm these findings (Figure 1). Notably, there were differences in cumulative incidence of osteoporosis in those below the 3rd, at the 50th through 84th, and above the 95th BMI percentiles already after approximately 5 years.

**Table 2.** The Association Between Adolescent BMI and Incident Osteoporosis^a^

Characteristic	Participants in each percentile, No. (%)						
	<3rd	3rd to 4th	5th to 9th	10th to 49th	50th to 84th	85th to 94th	≥95th
Men	30 201	14 608	33 842	247 801	202 605	54 081	31 446
Osteoporosis cases	460	213	467	3211	2012	415	151
Follow-up, mean (SD), y	18.7 (7.3)	19.0 (7.2)	18.8 (7.3)	18.6 (7.3)	17.7 (.6)	16.4 (7.7)	14.9 (7.5)
Cumulative follow-up, person-years	564 848	276 952	637 268	4 617 486	3 593 676	888 243	468 471
Incident rate, per 10^5^ person-years	81.4	76.9	73.3	69.5	56.0	46.7	32.2
Age at diagnosis, mean (SD), y	53.8 (12.5)	56.8 (10.9)	55.6 (12.1)	58.4 (10.9)	58.4 (11.1)	57.7 (11.2)	54.9 (13.1)
HR (95% CI)	1.82 (1.65-2.02)	1.54 (1.34-1.77)	1.42 (1.29-1.57)	1.21 (1.14-1.28)	1 [Reference]	1.06 (0.95-1.18)	1.17 (0.99-1.38)
*P* value	<.001	<.001	<.001	<.001	NA	.28	.06
Adjusted HR (95% CI)	1.82 (1.64-2.01)	1.50 (1.30-1.74)	1.41 (1.27-1.56)	1.19 (1.12-1.26)	1 [Reference]	1.04 (0.93-1.16)	1.14 (0.96-1.34)
*P* value	<.001	<.001	<.001	<.001	NA	.53	.14
Women	12 800	7635	19 744	190 915	180 027	43 720	14 066
Osteoporosis cases	757	437	1178	10 339	7520	1099	167
Follow-up, mean (SD), y	17.9 (7.2)	18.2 (7.2)	18.3 (7.2)	18.2 (7.3)	17.8 (7.5)	16.8 (7.6)	15.1 (7.5)
Cumulative follow-up, person-years	229 225	139 225	361 616	3 474 940	3 203 955	732 604	211 699
Incident rate, per 10^5^ person-years	330.2	313.9	325.8	297.5	234.7	150.0	78.9
Age at diagnosis, mean (SD), y	52.1 (8.5)	52.6 (8.6)	53.1 (8.1)	54.5 (7.9)	55.3 (7.6)	55.1 (8.3)	52.9 (9.3)
HR (95% CI)	1.87 (1.74-2.02)	1.63 (1.48-1.80)	1.59 (1.49-1.69)	1.26 (1.23-1.30)	1 [Reference]	0.83 (0.78-0.88)	0.83 (0.71-0.96)
*P* value	<.001	<.001	<.001	<.001	NA	<.001	.01
Adjusted HR (95% CI)	1.88 (1.74-2.04)	1.66 (1.50-1.84)	1.59 (1.49-1.69)	1.27 (1.23-1.32)	1 [Reference]	0.83 (0.77-0.89)	0.86 (0.74-1.01)
*P* value	<.001	<.001	<.001	<.001	NA	<.001	.06

Abbreviations: HR, hazard ratio; NA, not applicable.

^a^ Models were stratified by sex. Incident rate was calculated per 10^5^ person years. Models were adjusted for age at follow-up initiation, birth year, education, cognitive performance, socioeconomic status, and country of origin.

**Figure 2.**
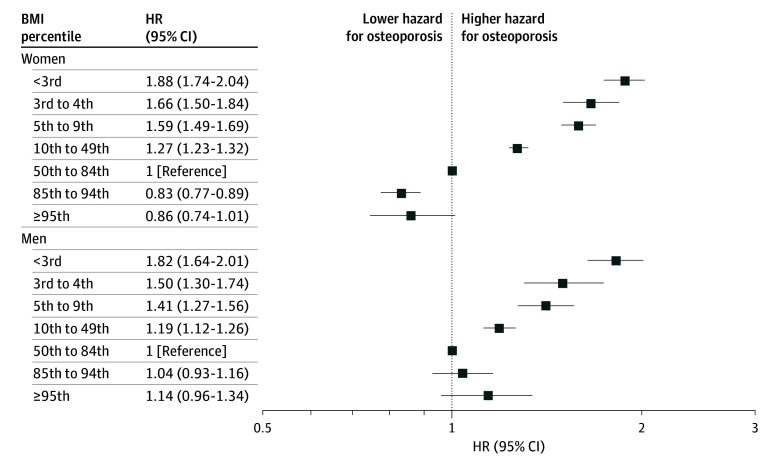
The Association Between Adolescent Body Mass Index (BMI) and Incident Osteoporosis Cox proportional hazard models are shown for women and men. Each analysis included 7 BMI categories (calculated as weight in kilograms divided by height in meters squared). Model was adjusted for age at follow-up initiation, birth year, education, cognitive performance, socioeconomic status, and country of origin. HR indicates hazard ratio.

Point estimates were overall consistent in unadjusted and adjusted models. Among women, adjusted HRs for osteoporosis incidence decreased gradually from low to high BMI values (Figure 2): the HR was 1.88 (95% CI, 1.74-2.04) for those below the 3rd percentile, 0.83 (95% CI, 0.77-0.89) for those with overweight and 0.86 (95% CI, 0.74-1.01) for those with obesity. Among men, a similar pattern was observed in the low BMI range below the 3rd percentile, with an HR of 1.14 (95% CI, 0.96-1.34) for those with obesity. For both sexes, we did not observe significant HRs when the obesity group was further subdivided into those with class I vs class II to III obesity (eTable 3 in Supplement 1). Results persisted when the study sample was limited to examinees with unimpaired health at baseline to minimize confounding by preexisting or coexisting morbidities (eTable 4 in Supplement 1). Moreover, results remained when people who were diagnosed with cancer or diabetes during follow-up were excluded from the analysis. Point estimates were accentuated for early-onset disease before 52 years old (eTable 5 in Supplement 1), or when analysis was limited to people who entered the study after 1995 (men) or 1996 (women) (eTable in Supplement 1). A sensitivity analysis that included only those who were MHS permanent members yielded consistent results (eTable 6 in Supplement 1).

### Adulthood Prediagnosis BMI and Osteoporosis Risk

**Figure 3.**
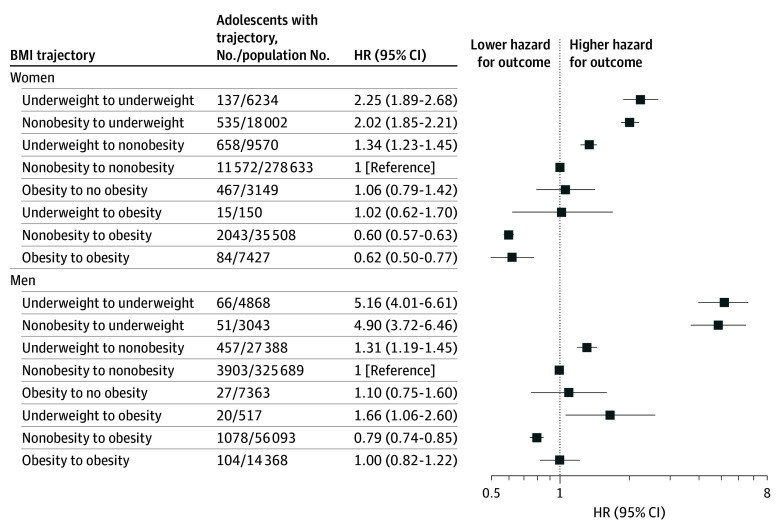
Osteoporosis Incidence According to Body Mass Index (BMI) Trajectories Forest plots among women and men present HRs and 95% CIs for incident osteoporosis among 9 BMI groups (calculated as weight in kilograms divided by height in meters squared) indicating BMI change from adolescent to prediagnosis measurement. The reference group was those with persistently nonobese BMI across the entire period. HR indicates hazard ratio.

Adulthood BMI data were available for 439 350 men (71.5%) and 358 721 women (76.5%). Baseline characteristics of this population vs those without adult BMI data (eTable 7 in Supplement 1) indicated no substantial differences in medical and sociodemographic indices, except for entering the study at earlier decades. The mean (SD) age of the second measurement was 34.8 (11.0) years for men (185 of 5706 osteoporosis cases occurred before that age) and 32.2 (10.4) years for women (161 of 15 096 osteoporosis cases occurred before that age). Weight gain between the 2 measurements was associated with reduced hazard ratios for osteoporosis incidence (Figure 3). However, weight loss was associated with increased hazards. This observation persisted when people who developed cancer (which may cause weight reduction and confound the association with osteoporosis) were excluded from analysis (eTable 8 in Supplement 1).

Women with adulthood BMI in the underweight range had the highest risk for osteoporosis, regardless of BMI status at adolescence; the underweight HR was 2.25 (95% CI, 1.89-2.68) and the nonobese HR was 2.02 (95% CI, 1.85-2.21), compared with the reference group of those with persistently nonobese BMI across the entire period (Figure 3 and eTable 8 in Supplement 1). A similar pattern was observed among men with even higher HRs; 5.16 for underweight (95% CI, 4.01-6.61) and 4.90 for nonobese (95% CI, 3.72-6.46). Small numbers of examinees in both sexes with adolescent obesity who became underweight at adulthood limited meaningful analysis. Women who were underweight at adolescence and nonobese BMI at adulthood had an HR of 1.34 (95% CI, 1.23-1.45) compared with women with sustained nonobese BMI (reference group). The HR of women with underweight at adolescence who developed obesity at adulthood was comparable with the reference group (HR, 1.02; 95% CI, 0.62-1.70). Women with sustained obesity from adolescence to adulthood had an HR of 0.62 (95% CI, 0.50-0.77).

## Discussion

In this nationwide retrospective cohort study of over 1 million participants with adolescent BMI measurements, we assessed the risk for osteoporosis in adulthood according to the adolescent BMI and the adolescent-adulthood BMI trajectory by sex. We report an adjusted HR of 1.88 and 1.82 among underweight women and men respectively, that monotonically decreased to 0.86 and 1.14 for those with obesity. Furthermore, a history of being underweight in adolescence nearly doubled the risk for osteoporosis in both sexes regardless of weight status in early adulthood.

There is ample evidence supporting the relationship between BMI and BMD, though the vast majority comes from studies in adult^6^ or elderly patients.^23^ Those studies, both cross-sectional^23^ and prospective,^24^ robustly support a protective effect of obesity on osteoporosis risk, albeit with a variable effect size. A meta-analysis^25^ of 121 studies (cross-sectional for BMD and prospective for fracture outcomes) demonstrated that obesity was associated with higher BMD and with hip fracture risk reduction of 41% and 25% in men and postmenopausal women, respectively. Another meta-analysis^26^ of 24 studies, most of which were cross-sectional, reported an odds ratio (OR) of 0.3 for incident osteoporosis among people with obesity vs normal weight. Of note, adolescents were not included, and young adults were underrepresented in both meta-analyses.

Some studies, however, challenged the linear nature of the BMI-osteoporosis relationship and suggested that at higher BMI values the risk is flattened.^24^ Shen et al,^27^ for example, showed a decreasing risk for hip fracture with increasing BMI, which plateaued just below a BMI of 30 in men but not in women. In agreement, we noted in both sexes comparable HRs among those with mild vs more advanced degrees of obesity. Our observation that adolescent obesity conferred a protective risk for osteoporosis in women but not in men likely arises from the sex-dependent effect of obesity on the gonadal axis. In men, obesity, particularly of visceral origin, induces androgen deficiency and results in male obesity-related secondary hypogonadism—a highly prevalent condition that ranges between 30% and 64% depending on the degree of obesity.^24,28,29,30^ Notably, visceral obesity, which is more prevalent in men, was linked to higher osteoporosis risk. Women with obesity, however, usually present with excess estrogen and possibly higher androgen levels.^28^ Although the relative importance of testosterone vs estrogen in male bone physiology is complex, we speculate that obesity-induced hypogonadism underlies the sex-dependent effect of obesity on osteoporosis risk. Other evidence also supports that delayed puberty may reduce bone accrual during critical growth periods, more so in males than females.^31^ Relevant hormonal levels were unavailable to us and preclude the confirmation of this hypothesis. Another possible explanation may arise from the inherent sex-dependent differences in bone geometry (smaller and thinner bones in females) and the positive effect of mechanical loading that may be more influential in people with less favorable bone geometry, especially during a peak bone mass accrual period.^32^

Conversely, low weight is a recognized risk factor for osteoporosis. One meta-analysis^26^ reported an OR of 2.54 for osteoporosis in people with underweight vs normal weight. Another meta-analysis^29^ of approximately 60 000 people reported an increased risk for osteoporosis of 2% per unit decrement in BMI with a much steeper gradient for BMI values lower than 20. Our results align with these point estimates and underscore underweight as a risk factor per se even among those with otherwise unimpaired health. Notably, underweight was linked to delays in every stage of puberty development in males^33^ and with delayed age at menarche in women.^34^ Thus, while delayed puberty may exert a positive effect on final height,^35^ it may be associated with persistently lower bone mineral density.^31^

Weight, among other factors, plays an important role in bone mass accrual during growth.^36^ Peak bone mass is an important determinant of bone health and is affected by nutritional, lifestyle, and metabolic factors, exercise, and coexisting morbidities in early life and adolescence.^36,37,38^ By the age of 18 roughly 90% of peak bone mass has been reached, with continued accruals up to the end of the second decade of life.^39^ Mechanical loading is essential during growth for the development of robust weight-bearing bones. In the absence of mechanical usage, limbs might underreach only 30% to 50% of normal bone mass, thereby leading to development of thin, fragile, long bones with diminished periosteal circumference. The highest osteoporosis risk in our study was recorded in those with sustained thinness from adolescence throughout adulthood. Moreover, adolescent underweight, irrespective of adult BMI, remained associated with nearly double the risk of osteoporosis into middle age. This cannot be explained by major chronic diseases such as cancer and diabetes, which may confound the BMI-osteoporosis association, but were controlled for here. These findings underscore the importance of the adolescence-to-adulthood transition time window for bone health. We are unaware of similar studies that focused on weight change in early adulthood, though evidence exists for middle-aged adults. In the Framingham cohort, 716 women and 448 men were followed up for 40 years. That study showed that low weight at baseline (mean age 36 years) was a strong risk factor for low BMD, as was weight change during study period, especially in men, and less so in women.^6^ With agreement, the Norwegian Epidemiological Osteoporosis study followed up 1497 men from mean age of 45 to 68 years and showed that a lower BMI at first measurement was associated with lower age-adjusted BMD regardless of weight change.^40^ It has also previously been estimated that a 10% increase in peak bone mass in the female population would be associated with a 50% decrease in the risk of fractures later in life.^36^ Thus, our findings collectively support a legacy effect of underweight at adolescence that contributes to a higher prevalence of osteoporosis in adulthood among both sexes.

### Limitations and Strengths

Our study has limitations. First, lifestyle data of the participants, including physical activity and diet, as well as family history of osteoporosis, were not available. We lacked information about use of medications that may affect osteoporosis risk. Notably, this drawback is mitigated by a sensitivity analysis restricted to those with unimpaired health at baseline. Since osteoporosis in adulthood was detected via dual-energy X-ray absorptiometry tests, some detection bias may be present if underweight participants were more likely to be referred to BMD assessments. Additionally, obesity and underweight status were determined according to BMI rather than waist circumference and other measures of adiposity. Fourth, only participants from MHS were included in this study. However, selection bias is not expected due to the National Health Insurance Law and free selection between HMOs, as well as previous studies based on this linkage that demonstrated high external validity.^41,42,43,44^ Finally, our cohort is underrepresentative for the Israeli Arab population and Orthodox Jewish women, who are not obligated to join the army.

The strengths of this study include its nationwide screening process, systematic medical evaluation, and data collection that were available for a large number of people and allowed us to divide BMI into multiple groups and examine the association with higher granularity. Also, a second prediagnosis BMI assessment was available for most of the study population with careful control on confounding morbidities such as diabetes or cancer.

## Conclusion

In conclusion, in this cohort study of 1 083 491 adolescents, we provided evidence for an association between BMI in adolescence and the risk of osteoporosis in adulthood, along with the associations of weight changes. This emphasizes the importance of healthy weight in adolescence for the prevention of osteoporosis among other diseases later in life.

## Notes

Supplement 1. **eTable 1. **Baseline Characteristics of MHS Members vs Other HMOs in Israel **eTable 2. **Baseline Characteristics of People Who Were Lost to Follow-Up **eTable 3. **Osteoporosis Incidence Among People With Obesity by Degree of Severity **eTable 4. **Various Sensitivity Analyses for the Association Between BMI and Osteoporosis **eTable 5.** A Subgroup Analysis for the Association Between BMI and Osteoporosis by Age of Study Outcome **eTable 6. **Sensitivity Analyses for People Who Were Permanently Members in MHS Throughout the Entire Study Period **eTable 7 **Baseline Characteristics of the Study Population According to Availability of Adulthood BMI Data **eTable 8. **BMI Trajectories From Adolescence to Adulthood and Osteoporosis Risk: Detailed Description of Model Results **eFigure. **Description of Study Cohort Build-Up

Supplement 2.
Data Sharing Statement
