# Supplementary material for: Adolescent Body Mass Index, Weight Trajectories to Adulthood, and Osteoporosis Risk
Source: JAMA Netw Open. 2025 Aug 4;8(8):e2525079. doi: 10.1001/jamanetworkopen.2025.25079 (PMC12322795; doi:10.1001/jamanetworkopen.2025.25079)
Supplement: Supplement 1. — eTable 1. Baseline Characteristics of MHS Members vs Other HMOs in Israel eTable 2. Baseline Characteristics of People Who Were Lost to Follow-Up eTable 3. Osteoporosis Incidence Among People With Obesity by Degree of Severity eTable 4. Various Sensitivity Analyses for the Association Between BMI and Osteoporosis eTable 5. A Subgroup Analysis for the Association Between BMI and Osteoporosis by Age of Study Outcome eTable 6. Sensitivity Analyses for People Who Were Permanently Members in MHS Throughout the Entire Study Period eTable 7 Baseline Characteristics of the Study Population According to Availability of Adulthood BMI Data eTable 8. BMI Trajectories From Adolescence to Adulthood and Osteoporosis Risk: Detailed Description of Model Results eFigure. Description of Study Cohort Build-Up [file jamanetwopen-e2525079-s001.pdf]

Supplemental Online Content

Simchoni M, Landau R, Derazne E, et al. Adolescent BMI, Weight Trajectories to Adulthood, and Osteoporosis Risk. *JAMA Netw Open*. 2025;8(7):e2525079. doi:10.1001/jamanetworkopen.2025.25079

eTable 1. Baseline Characteristics of MHS Members vs Other HMOs in Israel
eTable 2. Baseline Characteristics of People Who Were Lost to Follow-Up
eTable 3. Osteoporosis Incidence Among People With Obesity by Degree of Severity
eTable 4. Various Sensitivity Analyses for the Association Between BMI and Osteoporosis
eTable 5. A Subgroup Analysis for the Association Between BMI and Osteoporosis by Age of Study Outcome
eTable 6. Sensitivity Analyses for People Who Were Permanently Members in MHS Throughout the Entire Study Period
eTable 7 Baseline Characteristics of the Study Population According to Availability of Adulthood BMI Data
eTable 8. BMI Trajectories From Adolescence to Adulthood and Osteoporosis Risk: Detailed Description of Model Results
eFigure. Description of Study Cohort Build-Up

This supplemental material has been provided by the authors to give readers additional information about their work.

© 2025 Simchoni M et al. *JAMA Network Open*.

Israel

Characteristics at baseline after exclusions of those with missing BMI data or confounding comorbidities of MHS members vs. other HMO's in Israel who attend pre-recruitment medical evaluation prior to mandatory military service between 1967 to 2019.

	Maccabi Health Care services	Other health maintenance organizations
**Men**		
**Number**	614,584	1,115,217
**Mean BMI** (Kg/m^2 ±SD)	21.8±3.5	21.8±3.6
**Mean weight** (Kg ±SD)	66.4±12.0	65.8±12.4
**Mean height** (cm ±SD)	174.2±6.8	173.3±6.9
**Mean age at BMI measurement (**years ±SD)	17.3±0.5	17.4±0.5
**Blood pressure** (mmHg ±SD)		
Systolic	120.2±11.9	120.3±11.9
Diastolic	72.1±8.4	71.9±8.5
**Full education** (%)	87.5	80.7
**SES** (%)		
Low	21.6	33.2
Medium	50.2	50.2
High	28.2	16.5
**Cognitive score** (%)		
Low	13.7	20.6
Medium	68.0	66.3
High	18.3	13.2
**Israeli Born** (%)	81.9	83.6
**Unimpaired health** (%)	71.8	73.4
**Women**		
**Number**	468,907	706,327
**Mean BMI** (Kg/m^2 ±SD)	21.7±3.4	21.7±3.6
**Mean weight** (Kg ±SD)	57.2±9.9	57.4±10.5
**Mean height** (cm ±SD)	162.4±6.1	161.9±6.2
**Mean age at BMI measurement (**years ±SD)	17.3±0.4	17.3±0.5
**Blood pressure** (mmHg ±SD)		
Systolic	112.6±11.8	112.9±11.7
Diastolic	70.3±8.2	70.4±8.3
**Full education** (%)	96.0	94.3
**SES** (%)		
Low	15.6	24.5
Medium	52.5	55.4
High	31.9	20.1
**Cognitive score** (%)		
Low	9.5	13.8
Medium	75.2	73.7
High	15.3	12.5
**Israeli Born** (%)	83.9	86.7
**Unimpaired health** (%)	77.1	77.7

© 2025 Simchoni M et al. *JAMA Network Open*.

eTable 2. Baseline Characteristics of People Who Were Lost to Follow-Up

Follow-up	Completed follow-up	Lost to follow-up
**Men**		
**Number**	498,148	116,436
**Mean BMI** (Kg/m^2 ±SD)	21.8±3.5	21.9±3.5
**Mean weight** (Kg ±SD)	66.4±11.9	66.7±12.0
**Mean height** (cm ±SD)	174.2±6.8	174.3±6.8
**Mean age at BMI measurement (**years ±SD)	17.3±0.5	17.3±0.5
**Mean age at follow-up beginning** (years ±SD)	24.3±8.9	23.0±8.1
**Full education** (%)	87.5	87.5
**SES** (%)		
Low	20.7	25.2
Medium	50.4	49.5
High	28.8	25.4
**Cognitive score** (%)		
Low	13.7	13.8
Medium	68.6	65.9
High	17.8	20.3
**Israeli Born** (%)	82.7	78.7
**Women**		
**Number**	405,304	63,603
**Mean BMI** (Kg/m^2 ±SD)	21.7±3.4	21.7±3.4
**Mean weight** (Kg ±SD)	57.2±9.9	57.4±9.8
**Mean height** (cm ±SD)	162.4±6.1	162.5±6.1
**Mean age at BMI measurement (**years ±SD)	17.3±0.4	17.3±0.5
**Mean age at follow-up beginning** (years ±SD)	23.3±8.3	21.9±7.0
**Full education** (%)	96.1	95.7
**SES** (%)		
Low	15.0	19.3
Medium	52.5	52.8
High	32.5	27.9
**Cognitive score** (%)		
Low	9.4	10.6
Medium	75.4	73.4
High	15.2	16.0
**Israeli Born** (%)	84.6	79.2

© 2025 Simchoni M et al. *JAMA Network Open*.

eTable 3. Osteoporosis Incidence Among People With Obesity by Degree of Severity

Models were stratified by sex. Incident rate was calculated per 10^5 person years. Models were adjusted for age at follow-up beginning, birth year, education, cognitive performance, socioeconomic status, and country of origin. CI, confidence interval; HR, hazard ratio, SD, standard deviation.

	**Normal weight 50th to 84th**	**Overweight 85th to 94th**	**Obese Ⅰ 95th to 119% of 95th perc.**	**Obese Ⅱ+Ⅲ ≥120% of 95th perc.**
		**Men**		
**Number**	195,732	52,038	24,979	5,434
**Cumulative follow-up** (person-years)	3,460,908	853,447	380,667	72,728
**Osteoporosis cases**	1,918	392	136	12
**Incident rate** (per 10^5 person-years)	55.4	45.9	35.7	16.5
**Adjusted HR**<br>95% CI<br>P-value	Reference	1.04<br>0.93-1.16<br>0.50	1.16<br>0.98-1.38<br>0.09	0.99<br>0.56-1.75<br>0.98
		**Women**		
**Number**	173,162	41,948	10,884	2,764
**Cumulative follow-up** (person-years)	3,071,961	702,650	166,862	38,497
**Osteoporosis cases**	6,617	972	138	21
**Incident rate** (per 10^5 person-years)	215.4	138.3	82.7	54.5
**Adjusted HR**<br>95% CI<br>P-value	Reference	0.83<br>0.77-0.89<br><0.001	0.86<br>0.73-1.02<br>0.09	0.94<br>0.61-1.44<br>0.77

© 2025 Simchoni M et al. *JAMA Network Open*.

eTable 4. Various Sensitivity Analyses for the Association Between BMI and Osteoporosis

Models were stratified by sex. Incident rate was calculated per 10^5 person years. Models were adjusted for age at follow-up beginning, birth year, education, cognitive performance, socioeconomic status, and country of origin. CI, confidence interval; HR, hazard ratio, SD, standard deviation.

	<3rd perc.	3rd to 4th	5th to 9th	10th to 49th	50th to 84th	85th to 94th	≥95th
**Men**							
**Adolescents with unimpaired health**							
N	19,013	9,802	23,265	175,729	143,431	35,868	19,200
HR	1.83	1.48	1.29	1.21		1.05	1.04
95% CI	1.62-2.07	1.25-1.75	1.15-1.46	1.14-1.29	Reference	0.93-1.19	0.83-1.30
P-value	<0.001	<0.001	<0.001	<0.001		0.40	0.72
**Excluded cases of cancer or diabetes during follow-up**							
N	27,459	13,223	30,376	219,622	176,591	46,539	27,435
HR	1.92	1.51	1.53	1.16		1.04	1.03
95% CI	1.70-2.16	1.27-1.79	1.36-1.72	1.09-1.25	Reference	0.91-1.20	0.81-1.31
P-value	<0.001	<0.001	<0.001	<0.001		0.56	0.79
**Recent decades (pre-recruitment assessment ≥1995**)							
N	14,684	6,863	15,729	116,333	105,103	33,174	22,816
HR	3.28	2.04	2.08	1.44		0.81	1.23
95% CI	2.28-4.71	1.14-3.67	1.37-3.15	1.10-1.88	Reference	0.50-1.32	0.75-2.02
P-value	<0.001	0.01	<0.001	0.008		0.40	0.42
**Women**							
**Adolescents with unimpaired health**							
N	8,954	5,402	14,305	143,384	135,200	31,130	9,254
HR	1.89	1.67	1.56	1.27		0.80	0.89
95% CI	1.73-2.06	1.49-1.87	1.46-1.68	1.23-1.31	Reference	0.74-0.86	0.73-1.09
P-value	<0.001	<0.001	<0.001	<0.001		<0.001	0.27
**Excluded cases of cancer or diabetes during follow-up**							
N	11,530	6,831	17,570	168,347	157,284	38,001	12,432
HR	1.92	1.78	1.61	1.29		0.84	0.92
95% CI	1.76-2.09	1.59-1.99	1.50-1.73	1.24-1.34	Reference	0.78-0.91	0.76-1.11
P-value	<0.001	<0.001	<0.001	<0.001		<0.001	0.36
**Recent decades (pre-recruitment assessment ≥1996**)							
N	6,904	3,937	10,031	95,298	92,569	25,833	10,185
HR	3.26	3.37	2.50	1.66	Reference	1.00	0.89
95% CI	2.12-5.01	1.99-5.71	1.67-3.74	1.31-2.12		0.65-1.54	0.43-1.82
P-value	<0.001	<0.001	<0.001	<0.001		0.99	0.74

© 2025 Simchoni M et al. *JAMA Network Open*.

eTable 5. A Subgroup Analysis for the Association Between BMI and Osteoporosis by Age of Study Outcome

Models were stratified by sex Incident rate was calculated per 10^5 person years. Models were adjusted for age at follow-up beginning, birth year, education, cognitive performance, socioeconomic status, and country of origin. CI, confidence interval; HR, hazard ratio, SD, standard deviation.

	<3rd perc.	3rd to 4th	5th to 9th	10th to 49th	50th to 84th	85th to 94th	≥95th
**Men**							
**Osteoporosis diagnosis <52 years**							
N	21,876	10,254	23,639	171,176	146,967	42,835	27,171
HR	2.47	1.65	1.74	1.20		1.07	1.18
95% CI	2.07-2.93	1.27-2.16	1.45-2.10	1.06-1.34	Reference	0.87-1.32	0.89-1.57
P-value	<0.001	<0.001	<0.001	0.003		0.54	0.26
**Osteoporosis diagnosis ≥52 years**							
N	7,617	4,024	9,342	69,390	48,765	9,203	3,242
HR	1.60	1.49	1.32	1.20		1.02	1.10
95% CI	1.40-1.83	1.25-1.78	1.17-1.50	1.12-1.28	Reference	0.89-1.15	0.90-1.36
P-value	<0.001	<0.001	<0.001	<0.001		0.82	0.36
**Women**							
**Osteoporosis diagnosis <52 years**							
N	10,117	5,920	15,056	142,826	135,982	35,260	12,527
HR	2.43	2.06	1.93	1.39		0.74	0.79
95% CI	2.16-2.72	1.74-2.40	1.75-2.14	1.32-1.47	Reference	0.66-0.84	0.61-1.03
P-value	<0.001	<0.001	<0.001	<0.001		<0.001	0.08
**Osteoporosis diagnosis ≥52 years**							
N	2,368	1,528	4,164	41,968	37,180	6,688	1,121
HR	1.53	1.39	1.38	1.21		0.88	0.94
95% CI	1.37-1.70	1.22-1.60	1.27-1.50	1.17-1.26	Reference	0.81-0.95	0.77-1.15
P-value	<0.001	<0.001	<0.001	<0.001		<0.001	0.56

© 2025 Simchoni M et al. *JAMA Network Open*.

eTable 6. Sensitivity Analyses for People Who Were Permanently Members in MHS Throughout the Entire Study Period

Models were stratified by sex. Incident rate was calculated per 10^5 person years. Models were adjusted for age at follow-up beginning, birth year, education, cognitive performance, socioeconomic status, and country of origin. CI, confidence interval; HR, hazard ratio, SD, standard deviation.

	<3rd perc.	3rd to 4th	5th to 9th	10th to 49th	50th to 84th	85th to 94th	≥95th
**Men**							
**Number**	24,573	11,913	27,406	199,022	160,553	42,700	24,891
**Osteoporosis cases**	440	201	447	3,024	1,918	392	148
**Cumulative follow-up** (person-years)	499,734	245,057	562,805	4,042,744	3,117,376	768,855	406,879
**Incident rate** (per 10^5 person-years)	88.0	82.0	79.4	74.8	61.5	51.0	36.4
**Adjusted HR**<br>95% CI<br>P-value	1.81<br>1.63-2.01<br><0.001	1.49<br>1.29-1.73<br><0.001	1.41<br>1.27-1.56<br><0.001	1.19<br>1.12-1.26<br><0.001	Reference	1.03<br>0.93-1.15<br>0.563	1.13<br>0.95-1.33<br>0.163
**Women**							
**Number**	11,000	6,502	16,819	161,022	150,055	36,340	11,904
**Osteoporosis cases**	697	400	1,079	9,246	6,617	972	159
**Cumulative follow-up** (person-years)	208,042	125,739	326,983	3,112,909	2,844,911	650,203	190,451
**Incident rate** (per 10^5 person-years)	335.0	318.1	330.0	297.0	232.6	149.5	83.5
**Adjusted HR**<br>95% CI<br>P-value	1.88<br>1.74-2.03<br><0.001	1.66<br>1.50-1.84<br><0.001	1.58<br>1.48-1.69<br><0.001	1.27<br>1.23-1.31<br><0.001	Reference	0.83<br>0.77-0.88<br><0.001	0.86<br>0.73-1.00<br>0.052

© 2025 Simchoni M et al. *JAMA Network Open*.

eTable 7 Baseline Characteristics of the Study Population According to Availability of Adulthood BMI Data

Adolescents were classified as having unimpaired health if their medical assessment revealed absence of medical conditions requiring a chronic medical treatment and/or follow-up, and no history of cancer or major operations. Full education was defined as ≥11 years (the maximum possible at the pre-recruitment evaluation). SES was grouped into 3 categories low (<4), medium (5-7), and high (>8). Cognitive score was categorized into low (<-1 SD), medium (between -1 SD and +1 SD), and high (>+1 SD). * Indicates a significant difference with p<0.05. BMI, body mass index; SD, standard deviation; SES, socioeconomic status.

	With adulthood BMI data	Without adulthood BMI data	Total
**Number** (% men)	798,071 (55.1)	285,420 (61.9)	1,083,491 (56.7)
**Mean BMI** (Kg/m^2 ±SD)	21.7±3.5	21.9±3.5	21.8±3.5
**BMI group** (%)			
<3rd perc.	4.0	3.8	4.0
3rd to 4th	2.1	1.9	2.1
5th to 9th	5.0	4.7	4.9
10th to 49th	40.9	39.4	40.5
50th to 84th	35.1	35.9	35.3
85th to 94th	8.8	9.6	9.0
≥95th perc.	4.1	4.6	4.2
**Systolic / Diastolic Blood pressure** (%) (mmHg ±SD)			
<120 / <80	43.6	43.1	43.5
≥120 / <80	15.9	18.8	16.7
≥130 / ≥80	34.6	32.6	34.1
≥140 / ≥90	5.8	5.6	5.8
**Unimpaired health** (%)	73.8	75.0	74.1
**Full education** (%)	91.0	91.8	91.2
**SES** (%)			
Low	18.5	20.5	19.0
Medium	51.8	49.6	51.2
High	29.7	29.9	29.8
**Cognitive score** (%)			
Low	11.6	12.7	11.9
Medium	71.8	69.2	71.1
High	16.6	18.0	17.0
**Israeli Born** (%)	82.1	84.8	82.8
**Decade of assessment** (%)			
1967-1979	10.5	7.3	9.7
1980-1989	18.2	10.0	16.1
1990-1999	26.3	22.8	25.4
2000-2009	24.7	12.6	21.6
2010-2018	20.3*	47.3*	27.3

© 2025 Simchoni M et al. *JAMA Network Open*.

eTable 8. BMI Trajectories From Adolescence to Adulthood and Osteoporosis Risk: Detailed Description of Model Results

Models were stratified by sex (men [A] and women [B]) and assessed the outcome of osteoporosis, among the nine BMI groups indicating BMI change from adolescent to pre-diagnosis measurement. Incident rate was calculated per 10^5 person years. The first model ('unadjusted') was only adjusted for age at follow-up beginning, and the multivariable models were also adjusted for birth year, education, cognitive performance, socioeconomic status, and country of origin. CI, confidence interval; HR, hazard ratio, SD, standard deviation. [detailed values for Figure 3].

**(A)**	**Under-Under**	**Under-Normal**	**Under-Obese**	**Normal-Under**	**Normal-Normal**	**Normal-Obese**	**Obese-Under**	**Obese-Normal**	**Obese-Obese**
**Number**	4,868	27,388	517	3,043	325,689	56,093	21	7,363	14,368
**Mean age at second BMI measurement** (years ±SD)	26.2±7.7	36.6±10.2	43.6±9.0	26.9±8.7	34.2±10.9	40.6±10.0	30.6±8.0	27.1±8.1	29.5±9.5
**Cumulative follow-up** (person-years)	43,486	282,976	5,813	26,173	3,231,243	660,470	190	61,334	137,998
**Osteoporosis**									
- Cases	66	457	20	51	3,903	1,078	0	27	104
- Incident rate	151.8	161.5	344.1	194.9	120.8	163.2	0	44.0	75.4
- Mean age at diagnosis	46.0±14.1	57.8±10.0	64.6±3.5	49.4±15.0	59.7±10.0	61.6±7.4	N/A	53.8±12.7	57.4±11.7
**Unadjusted HR**	5.31	1.31	1.61	4.79		0.79		1.06	1.02
- 95% CI	4.16-6.78	1.19-1.45	1.04-2.50	3.63-6.31	Reference	0.74-0.85	N/A	0.73-1.55	0.84-1.24
- P-value	<0.001	<0.001	0.03	<0.001		<0.001		0.76	0.85
**Adjusted HR**	5.16	1.31	1.61	4.90		0.79		1.10	1.00
- 95% CI	4.01-6.62	1.19-1.45	1.06-2.60	3.72-6.46	Reference	0.74-0.85	N/A	0.75-1.60	0.82-1.22
- P-value	<0.001	<0.001	0.03	<0.001		<0.001		0.63	0.99
**Excluded cases of cancer or diabetes during follow-up**									
- Number	4,697	24,400	324	2,898	289,147	37,895	0	6,739	11,525
- Adjusted HR	5.14	1.34	2.12	5.05		0.79		1.14	0.95
- 95% CI	3.97-6.67	1.20-1.50	1.23-3.67	3.79-6.75	1	0.72-0.87	N/A	0.71-1.84	0.70-1.28
- P-value	<0.001	<0.001	0.007	<0.001		<0.001		0.59	0.73

© 2025 Simchoni M et al. *JAMA Network Open*.

eTable 8. BMI Trajectories From Adolescence to Adulthood and Osteoporosis Risk: Detailed Description of Model Results

(B)	Under-Under	Under-Normal	Under-Obese	Normal-Under	Normal-Normal	Normal-Obese	Obese-Under	Obese-Normal	Obese-Obese
**Number**	6,234	9,570	150	18,002	278,633	35,508	48	3,149	7,427
**Mean age at second BMI measurement** (years ±SD)	26.1±7.2	35.5±9.9	42.2±10.0	27.8±8.1	32.1±10.3	37.4±10.3	29.9±8.9	27.4±8.0	26.5±8.0
**Cumulative follow-up** (person-years)	60,541	101,303	1,630	179,309	2,849,512	428,381	506	29,419	72,733
**Osteoporosis**									
- Cases	137	658	15	535	11,572	2,043	5	47	84
- Incident rate	226.3	649.5	920.2	298.4	406.1	476.9	988.6	159.8	115.5
- Mean age at diagnosis	47.2±10.4	55,5±7.1	57.0±5.4	50.4±9.4	56.5±7.0	58.1±6.3	49.9±11.3	55.1±7.0	53.1±9.6
**Unadjusted HR**	2.16	1.33	0.96	1.93		0.65	5.15	1.02	0.65
- 95% CI	1.82-2.56	1.23-1.44	0.58-1.59	1.77-2.11	Reference	0.62-0.68	2.14-12.37	0.76-1.35	0.52-0.80
- P-value	<0.001	<0.001	0.88	<0.001		<0.001	<0.001	0.91	<0.001
**Adjusted HR**	2.25	1.34	1.02	2.02		0.60	5.12	1.06	0.62
- 95% CI	1.89-2.68	1.23-1.45	0.62-1.70	1.85-2.21	Reference	0.57-0.63	2.13-12.31	0.79-1.42	0.50-0.77
- P-value	<0.001	<0.001	0.93	<0.001		<0.001	<0.001	0.72	<0.001
**Excluded cases of cancer or diabetes during follow-up**									
- Number	5,849	8,461	116	16,759		26,225	40	2,868	6,315
- Adjusted HR	2.36	1.39	1.28	2.01	248,283	0.61	6.18	1.06	0.65
- 95% CI	1.96-2.84	1.27-1.52	0.72-2.25	1.82-2.21	1	0.57-0.65	2.57-14.86	0.76-1.47	0.49-0.86
- P-value	<0.001	<0.001	0.40	<0.001		<0.001	<0.001	0.74	0.003

© 2025 Simchoni M et al. *JAMA Network Open*.

eFigure. Description of Study Cohort Build-Up

Confounding comorbidities excluded from the cohort: thyroid disorders, psoriasis, vitiligo, rheumatic autoimmune joint disorders, connective tissue diseases, vasculitis, Familial Mediterranean Fever, rheumatic fever, Raynaud's phenomenon, Celiac disease, inflammatory bowel disease, parathyroid and calcium homeostasis disorders, and childhood malignancies.

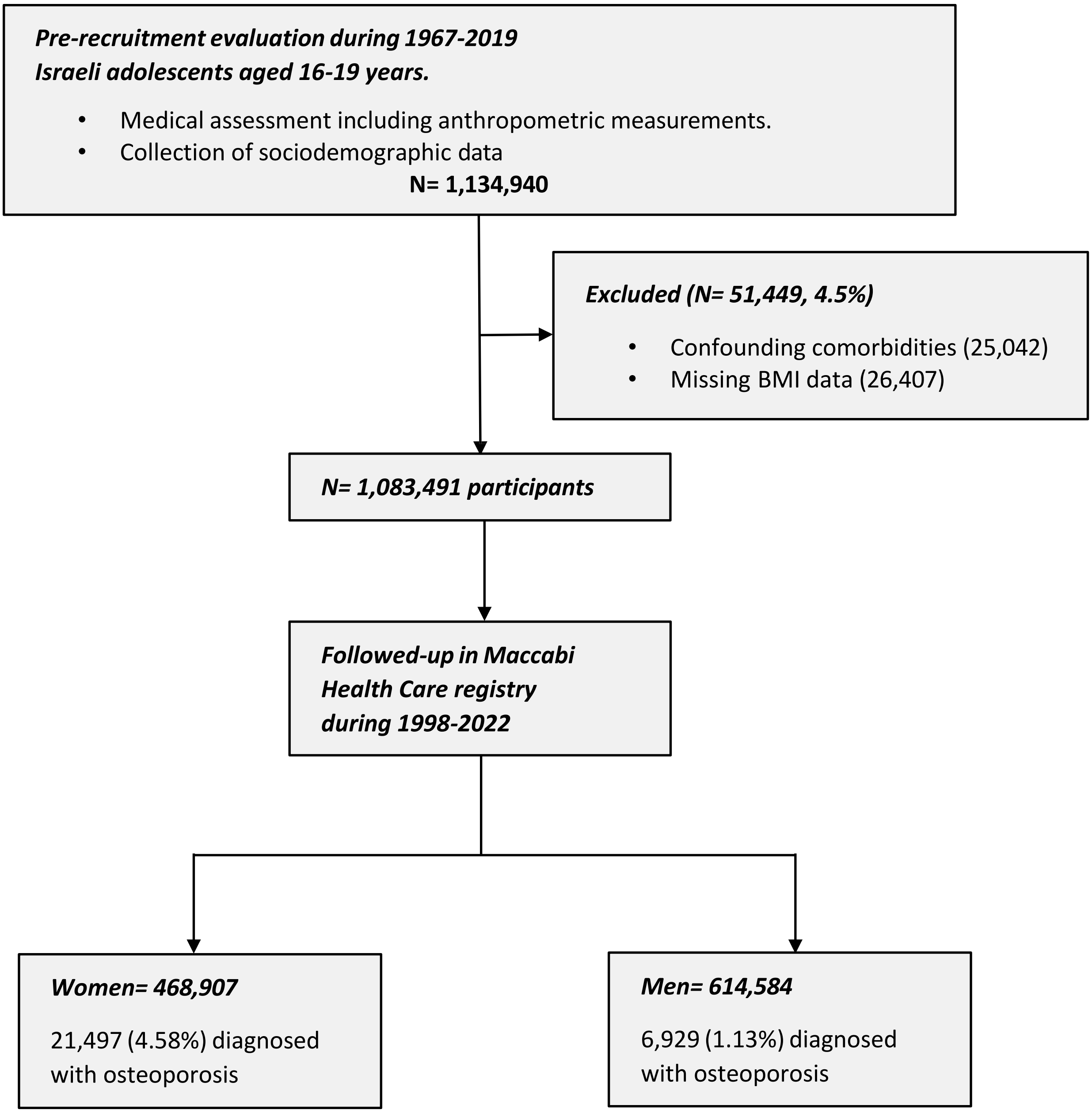

© 2025 Simchoni M et al. *JAMA Network Open*.
