# Supplementary material for: Adolescent Body Mass Index, Weight Trajectories to Adulthood, and Osteoporosis Risk
Source: JAMA Netw Open. 2025 Aug 4;8(8):e2525079. doi: 10.1001/jamanetworkopen.2025.25079 (PMC12322795; doi:10.1001/jamanetworkopen.2025.25079)
Supplement: Supplement 2. — Data Sharing Statement [file jamanetwopen-e2525079-s002.pdf]

Data Sharing Statement

Simchoni. Adolescent Body Mass Index, Weight Trajectories to Adulthood and Osteoporosis Risk. *JAMA Netw Open*. Published August 04, 2025.
doi:10.1001/jamanetworkopen.2025.25079

Data
Data available: No
